# Improved Deep CNN with Parameter Initialization for Data Analysis of Near-Infrared Spectroscopy Sensors

**DOI:** 10.3390/s20030874

**Published:** 2020-02-06

**Authors:** Di Wang, Fengchun Tian, Simon X. Yang, Zhiqin Zhu, Daiyu Jiang, Bin Cai

**Affiliations:** 1 School of Information Science and Engineering, Chongqing Jiaotong University, Chongqing 400074, China; diwang871106@gmail.com; 2 School of Microelectronics and Communication Engineering, Chongqing University, Chongqing 400044, China; 3 School of Engineering, University of Guelph, Guelph, ON N1G 2W1, Canada; 4 College of Automation, Chongqing University of Posts and Telecommunications, Chongqing 400065, China; zhuzq@cqupt.edu.cn (Z.Z.); daiyuj93@gmail.com (D.J.); 5 Guizhou Tobacco Rebaking Co. LTD, Guizhou 550025, China; cai.jiming2008@163.com

**Keywords:** NIR sensor, data analysis, convolutional neural network, cultivation region discrimination

## Abstract

Near-infrared (NIR) spectral sensors can deliver the spectral response of light absorbed by materials. Data analysis technology based on NIR sensors has been a useful tool for quality identification. In this paper, an improved deep convolutional neural network (CNN) with batch normalization and MSRA (Microsoft Research Asia) initialization is proposed to discriminate the tobacco cultivation regions using data collected from NIR sensors. The network structure is created with six convolutional layers and three full connection layers, and the learning rate is controlled by exponential attenuation method. One-dimensional kernel is applied as the convolution kernel to extract features. Meanwhile, the methods of L2 regularization and dropout are used to avoid the overfitting problem, which improve the generalization ability of the network. Experimental results show that the proposed deep network structure can effectively extract the complex characteristics inside the spectrum, which proves that it has excellent recognition performance on tobacco cultivation region discrimination, and it also demonstrates that the deep CNN is more suitable for information mining and analysis of big data.

## 1. Introduction

As the quality and flavor of tobacco leaf is affected heavily by the cultivation region, identification of tobacco origin plays a significant role before putting them into products [1]. In practical application, growing region discrimination is usually operated by trained experts through sensory inspection involving sense of smell, taste, and so on. However, the manual evaluation is very time-consuming, laborious, and depends on the experience of experts to a great extent. Such an evaluation cannot meet the requirement of reproducible assessment process for tobacco quality control and supervision. Therefore, an efficient and intelligent evaluation approach based on the near-infrared (NIR) data analysis is highly required for the tobacco industry.

As the advancement of spectral sensor technology, the applications of NIR sensors have been widely used for classification in many other fields: Yasmin et al. [2] applied a classification method to viability screening of naturally aged watermelon seeds using FT-NIR spectroscopy; Kim et al. [3] proposed finger-vein and finger shape multimodal biometrics using NIR light camera sensor based on a deep convolutional neural network (CNN); Manattayil et al. [4] studied nondestructive classification of diversely stained capsicum annuum seed specimens in different cultivars using NIR imaging based optical intensity detection; Nguyen et al. [5] proposed a PAD method for NIR camera-based finger-vein recognition system using CNN to enhance the detection ability of previous handcrafted methods; Lee et al. [6] proposed a CNN-based method for emotion detecting to identify aggressive driving using input images of the driver’s face that are obtained using NIR light and thermal camera sensors; Xiao et al. [7] investigated spectral analysis and sensitive waveband determination on nitrogen detection of different soil types with NIR sensors.

Meanwhile, the analysis technics of hyperspectral imaging combined with new intelligent algorithms can be also applied to classification [8,9,10]. The spectra acquired from NIR sensor have the potential to extract corresponding feature information of samples. Nowadays, the NIR spectral sensor technology has developed quickly as a powerful analytical method in many fields and proven the availability of the qualitative data analysis with different algorithms.

A lot of research works have been conducted on the quality analysis and quantity analysis of tobacco samples with different machine learning methods for data from NIR sensors [11,12,13]. Wang et al. [14] proposed least angle regression (LAR) to predict the content of chemical compositions, and results show that the LAR model is much superior to the least squares support vector machine (LS-SVM) model and partial least squares (PLS) model on prediction accuracy and operational efficiency; Wang et al. [15] also applied an SVM model to discriminate the tobacco cultivation region using the NIR sensors and adopted genetic algorithm for input subset selection to identify the effective principal components for the SVM model; Zhang et al. [16] proposed SVM to classify the production year of tobacco and obtained high accuracy. Duan et al. [17] established PLS regression for quantitative analysis of 27 chemical components for Chinese southwest tobacco. Da et al. [18] applied the mixed algorithm of PLS and ANN for the quantitative analysis of the total sugar in tobacco samples. Bin et al. [19] proposed a modified random forest approach to improve multi-class classification performance of tobacco leaf grades. 

In recent years, the deep structure of the neural network learning algorithm (usually with multiple hidden layers) is a hot spot for researchers, and it has shown great advantages in big data processing [20,21]. Especially, it has made a breakthrough in two-dimensional data (such as images) processing. NIR data has wide spectral band, serious overlap of spectral peak, and complex internal information characteristics, thus deep training is more suitable than shallow network (normally with one hidden layer) in processing complex information [22,23,24].

Deep CNN is a deep network structure, and it can extract high-level features from data through constructing multi-layer network structure, meaning it has better robustness and prediction performance than shallow network. There are many research works on applications of CNN to various problems using NIR data, e.g., Chen and Wang [25] proposed a CNN-based feature selection pruning and applied it to calibration modeling for NIR spectroscopy; Acquarelli et al. [26] applied a simple CNN architecture with a single convolutional layer to classify vibrational spectroscopic data and identify important spectral regions; Cui and Fearn [27] proposed a CNN for multivariate regression with applications to NIR calibration; Lee et al. [28] adopted deep residual CNN-based ocular recognition with rough pupil detection in the images by NIR camera sensors; Tazim et al. [29] investigated biometric authentication with CNN features of dorsal vein pattern extracted from NIR images. These research works show that CNN can be successfully applied to NIR sensor data processing. 

In the tobacco industry, especially for the classification problem of tobacco cultivation region, there are only few reports. Wang et al. [12] carried out tobacco quality analysis of different producing areas with spectrum projection and correlation methods, but only 1276 tobacco samples were used in their study. Lu et al. [30] improved the CNN classical model Lenet-5 with one-dimensional vector as the convolution kernel and single-layer sensing machines in C5, F6, and output layers of the lenet-5 structure. However, only 600 samples were used for the training and testing [30], which obviously is not enough for a CNN model that has many more parameters to be determined/trained using the known samples. In this paper, the deep CNN model is proposed in an innovative application to the classification of tobacco cultivation region for NIR data, where six convolution layers and three fully connected layers were established for CNN structure and one-dimensional vectors as convolution kernel were applied to extract complex features from data. To improve the generalization performance of the network, batch normalization was adopted in the convolution layers and the dropout technology was used in the fully connected layers. To avoid the phenomenon of gradient disappearance or explosion in the process of training network, the initialization for the weights of each layer of the network with Gaussian distribution of specific variance (MSRA initialization) was applied, which also accelerates the convergence of the model. In the proposed algorithm, the ReLU function was selected as the activation function of the network, and the cross-entropy function was chosen as the loss function. 

## 2. Materials and Methods

This section introduces the CNN model and relative methods, and describes the data source used in this study. Then, the evaluation criteria are given to conclude.

### 2.1. Convolutional Neural Network Model

A convolutional neural network is a deep neural network of supervised learning, and its structure includes convolution layers, pooling layers, and fully connected layers, among which the convolution layer and pooling layer are core parts to realize feature extraction of CNN [21,31]. The structure is first arranged alternately by convolution layer and pooling layer to achieve the extraction and mapping of local features from the NIR data, then successively arranged by several fully connected layers, and finally realized the classification of recognition targets by softmax. 

The core ideas of CNN are the local receptive field, weight sharing, and pooling layer, which greatly reduce the number of parameters in the neural network and effectively alleviates or avoids the overfitting phenomenon in the network model. Gradient descent method is adopted in CNN to minimize the loss function, and the weight parameters of the network is reversely adjusted, in which way the identification accuracy of the CNN model is improved through much iterative training [32]. 

As the NIR data is one-dimensional, the convolution kernel adopts one-dimensional vector to extract features from data. As the convolution kernel with small size and deep depth has the same effect as the convolution kernel with large size and shallow depth, but the parameters of the former are far less than that of the latter, the convolution kernel vector with size of 1 × 9 is firstly adopted to quickly obtain rough feature information, and then the convolution kernel vector with small size of 1×3 is used to extract more subtle data features. The method of full zero padding and upward rounding are adopted in the process of convolution. 

The pooling layer is used to reduce the size of feature map, and the maximum operation is used. The filter size of in the pooling layer is set as 1 × 2 in this study, and the step is set as 2. 

#### 2.1.1. Batch Normalization

Batch normalization (BN) [33] is a method that attempts to prevent overfitting. The batch standardized processing is applied before the activation function of each layer to make the output of the inactive follow a normal distribution with mean of 0 and variance of 1. Then, the results of batch standardized calculation are restored to the original input characteristics by zooming and panning. This process can ensure the capacity of the network, accelerate the training speed of network, and improve the generalization ability of the network [34,35]. In this paper, the batch size is set to be 16.

#### 2.1.2. Classifier and Loss Function

The function of softmax is used for neural network of multiple classification, and it is defined as

(1) $$S_{i}=\frac{e^{V_{i}}}{\sum_{i=1}^{C}e^{V_{i}}}$$

where *i* represents the category of data (there are eight classes in this study), $S_{i}$ is the probability of input with class i, $V_{i}$ is the output of the *i*-th node in the output layer, and C is the total number of categories. 

The function of cross entropy is the loss function in this study, which is used to describe the distance between two probability distributions, and it is given as

(2) $$L=-\sum_{i=1}^{C}p(i)\log(S_{i})$$

where p(i) is the real distribution of sample with class *i*. As one-hot coding is applied for data labels, p(i) equals to be one when the actual category of data is class *I*; otherwise, it iszero. Therefore, the loss function can be reduced as

(3) $$L=-\log(S_{i})$$

To reduce the noise in the training set, the regularization method of L2 is applied in loss function, and the loss function here is the sum of loss of the cross entropy and the loss of regularization.

#### 2.1.3. Activation Function

The function of rectified linear unit (ReLU) [36,37,38] is applied in this study, which makes the network have the nonlinear factor, and it is defined as

**Figure 1:**
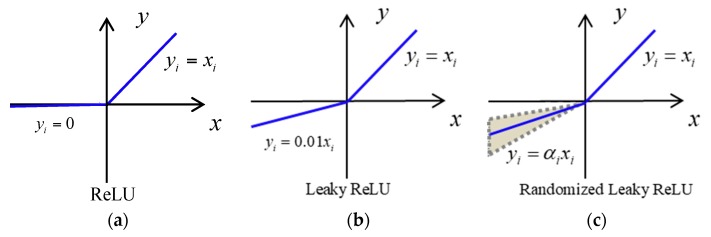
ReLU activation function and its variants. (**a**) ReLU function; (**b**) Leaky ReLU function; (**c**) Randomized Leaky ReLU function.

(4) f(x)=max(0,x)

where x is the input of the neural network. The ReLU function has two variants—leaky ReLU and Randomized Leaky ReLU—which are shown in Figure 1.

#### 2.1.4. Dropout

**Figure 2:**
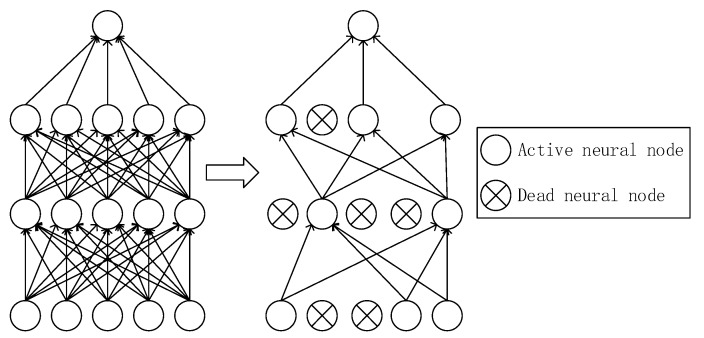
Illustration of dropout.

To prevent overfitting problems, dropout technology [39,40] is used on the fully connected layer. Dropout is used to make neurons stop working with a certain probability in each training batch, which means it makes the values of activation function turn to be zero with the probability. The probability is set to be 0.5 in this study, which means it makes half of nodes in fully connected layer stop working. The illustration of dropout is shown in Figure 2.

#### 2.1.5. Weight Initialization

As the formula of gradient contains the product of the weights of the subsequent layers and the derivative of the activation function, it may cause problem of gradient vanishing or gradient explosion, which makes the model unstable. To address this problem, the initialization method of MSRA (Microsoft Research Asia, also called Kaiming initialization or Delving Deep into Rectifiers) [41] for weights of each layer of the network is used. This initialization is a gaussian distribution with mean of 0 and variance of $\frac{2}{n}$ (n is the size of the convolution kernel of the layer), which is given as

(5) $$W\sim G(0,\sqrt{\frac{2}{n}})$$

### 2.2. Tobacco Database

**Figure 3:**
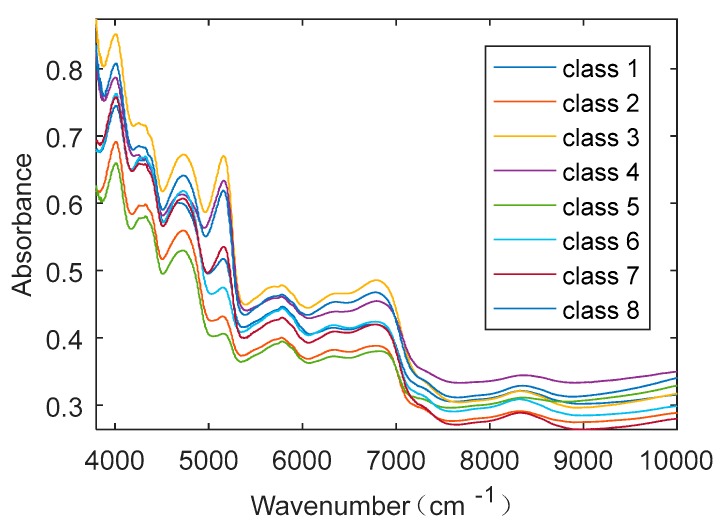
Raw near-infrared (NIR) spectra of 8 samples from 8 different regions.

A total of 13,370 tobacco NIR data were collected from eight different regions in Guizhou Province. The NIR spectra were recorded with Thermo Antaris 2 with multiple sensors (Thermo Fisher Scientific Inc. Waltham, USA). The spectra are with the resolution of 8 cm^−1^ and 64 scans. The NIR range is from 3800 cm^−1^ to 10,000 cm^−1^. Figure 3 shows the spectra of eight samples from eight different regions, and Table 1 shows the regional distribution of all the samples. The spectra were saved by the NIR sensor as digital data with absorbance values at different wavelength points of each sample, and the digital data were used as inputs of the clarification model in this study. Normally there are two ways to divide the samples available: One way is to divide the datasets randomly into a training set, validation set, and testing set, in which the training set is used to train the model, the validation set is used to verify the model performance during the training process and adjust some model parameters if needed, and the testing set are the unexposed samples that are used to check the recognition performance of the model after the model is well trained. Another way is to divide the datasets randomly into training set and testing set only, in which the training set is used to train the model and also adjust some model parameters during the training process, and the testing set with unexposed samples is used to check the recognition performance of the model after the model is well trained. In general, the first method requires more samples of data, and the training method is more complicated. The second way is simpler and more efficient, and it has demonstrated to be effective from our previous research works with proper training methods (see, e.g., in [14,15,42]). Thus, we chose the second way in this study, where the samples were randomly divided into training set (80%) and testing set (20%).

To study the recognition performance of CNN on different sample sets, and also to compare the CNN recognition performance with that of other traditional algorithms, a small sample data with 500 NIR data was adopted in this study. The small sample data were collected from four different regions in Guizhou Province with NIR range from 3499 cm^−1^ to 12004 cm^−1^. The small sample data were collected in 2008, and it is unfortunate that no additional samples can be added to the small sample data, as the type of NIR instrument used to obtain the small sample data was no longer available in our study.

Because CNN is a deep learning algorithm, the spectral feature at each wavelength point may be related to origin classification, and each convolution process is a process of extracting features with different information, so the original data is analyzed directly as input instead of dimensionality reduction and denoising.

### 2.3. Model Evaluation

The prediction accuracy is the significant parameter to evaluate the overall performance in the classification of the tobacco cultivation region, and it is defined as

(6) $$P_{a}=\frac{n_{r}}{N_{t}}$$

where $n_{r}$ is the number of samples predicted rightly and $N_{t}$ is the number of samples for prediction. In this study, $N_{t}$ is set to be 10,696 and 2674 in the training set and testing set, respectively.

One two-by-two confusion matrix, shown in Table 2, supports the evaluation criteria for the models. As shown in Table 2, *n* means the number of samples, and the parameters are defined according to the styles of the given label and the predicted label, where $n_{TP}$ is the number of positive samples that are labeled as positive, $n_{TN}$ is the number of negative samples labeled as negative, $n_{FP}$ is the number of positive samples labeled as negative, and $n_{FN}$ is the number of negative samples labeled as positive. The functions of the evaluation criteria are given as

(7) $$\gamma_{TP}=\frac{n_{TP}}{n_{TP}+n_{FN}}$$

(8) $$\gamma_{TN}=\frac{n_{TN}}{n_{TN}+n_{FP}}$$

(9) $$\gamma_{PP}=\frac{n_{TP}}{n_{TP}+n_{FP}}$$

(10) $$\gamma =\frac{2n_{TP}}{2n_{TP}+n_{FP}+n_{FN}}$$

where $\gamma_{TP}$ is the sensitivity rate, which is a measure of the ability to detect the positive patterns; $\gamma_{TN}$ is the specificity rate, which is means the ability to specify the negative patterns; $\gamma_{PP}$ is the precision rate, which represents the ability to predict the positive patterns; and γ is the F1-score, which considers both the precision and sensitivity of the test.

## 3. Results and Discussion 

**Figure 4:**
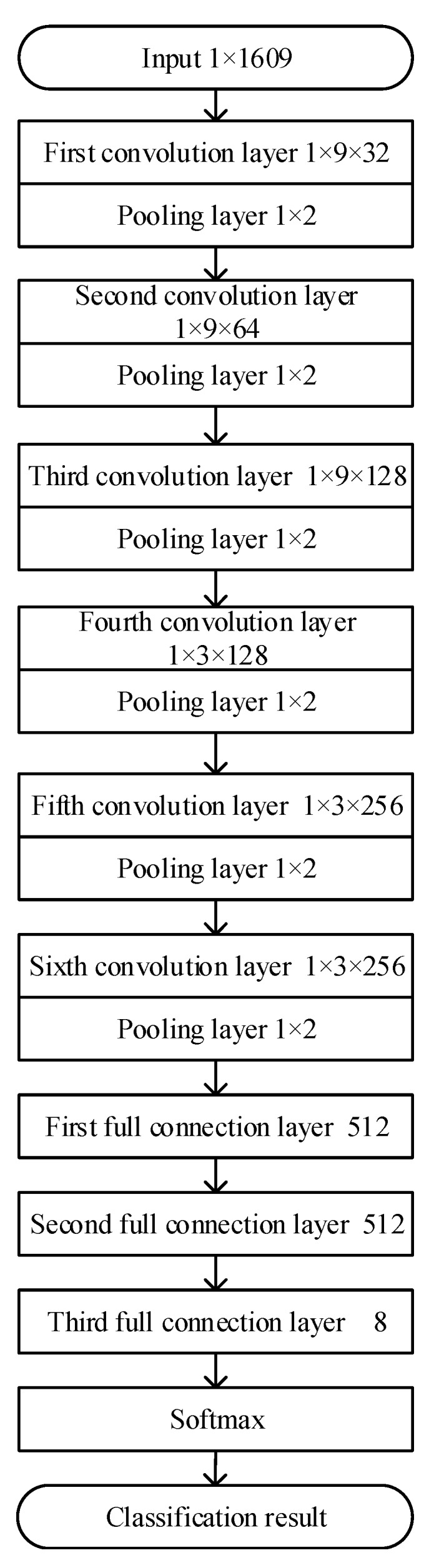
Schematic diagram of convolutional neural network (CNN) based on batch normalization.

The CNN model based on BN and MSRA initialization is applied in this study, and its structure is shown in Figure 4. First, six convolution layers are used to extract the input features and one maximum pooling layer is added after each convolution layer to reduce the number of parameters. Then, three full connection layers are used to conduct more advanced abstraction of the features. Finally, the softmax function is used to classify the output. To accelerate the training speed and improve the generalization ability of the network, the BN method is adopted to process the original input data and the data before the activation function after convolution operation in each convolution layer. The original data are one-dimensional NIR data and each dimension has 1609 features, therefore the convolution kernel in all convolution layers is set to be one-dimensional vector. 

As the effect of small size and deep convolutional kernel is the same as that of large size and shallow convolutional kernel, the parameters of the former are much less than those of the latter. To balance the number of parameters and the convolution speed, the 1 × 9 one-dimensional vector is used for the convolutional kernel in the first three convolution layers to quickly extract local features, and the 1 × 3 one-dimensional vector is used for the convolutional kernel in the latter three convolution layers to extract subtle features. To extract more subtle and different features, the channel number of the convolution kernel increases layer by layer, from 32 channels in the first convolution layer to 256 channels in the sixth convolution layer. The filter with size of 1 × 2 is adopted for all windows in the pooling layers. For the first two full connection layers, 512 nodes are adopted. As there are eight categories for the samples, eight nodes are used in the last full connection layer, and data classification results are obtained through the softmax function at the last layer. 

### 3.1. Setting of the Learning Rate

**Figure 5:**
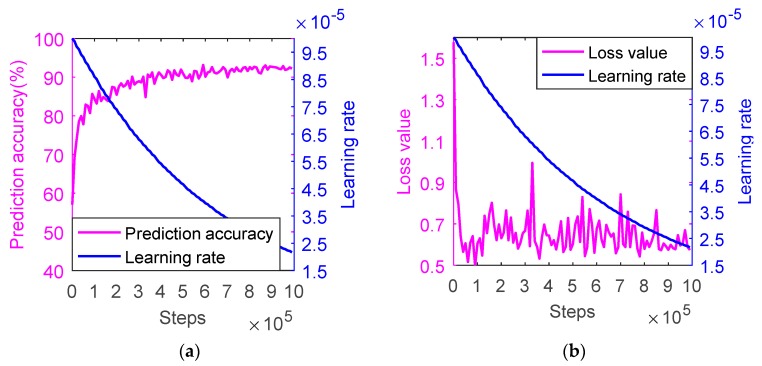
Setting of learning rate with exponential decay method using the training set. (**a**) The prediction accuracy v.s. the learning rate; (**b**) The loss value v.s. the learning rate.

When adopting the backpropagation algorithm, the CNN model will fail to converge if the learning rate is set too high, but it will easily fall into the local minimum if the learning rate is too small. In this study, exponential decay method is used to control the learning rate; the experimental results are shown in Figure 5.

In the experiment, the initial learning rate is 0.01 and the attenuation coefficient is 0.99. As seen in the figure, the blue curve represents the learning rate, and it shows obvious decline trend, which is because the algorithm obtains a relatively optimal solution by the learning rate of 0.01, then the learning rate is in accordance with the exponential decrease gradually along with the iterative progress, and it drops to 0.0000242 at the training round of 990,000. 

It can be seen from Figure 5a that when the learning rate is large, the prediction accuracy increases rapidly from 57%, and goes up to 82.9% at 6000 rounds, then it appears a trend of slow and steady rise with the decline of learning rate, and achieves 92.2% at 990,000 rounds. 

It can be seen from Figure 5b that with the decrease of learning rate, the loss value first drops rapidly from 1.577 at the beginning, decreases to 0.496 at 9000 rounds, then it gradually decreases in a wave-like shape after a small rise, and decreases to 0.573 at 990,000 rounds.

### 3.2. Setting of Number of Layers of CNN

**Figure 6:**
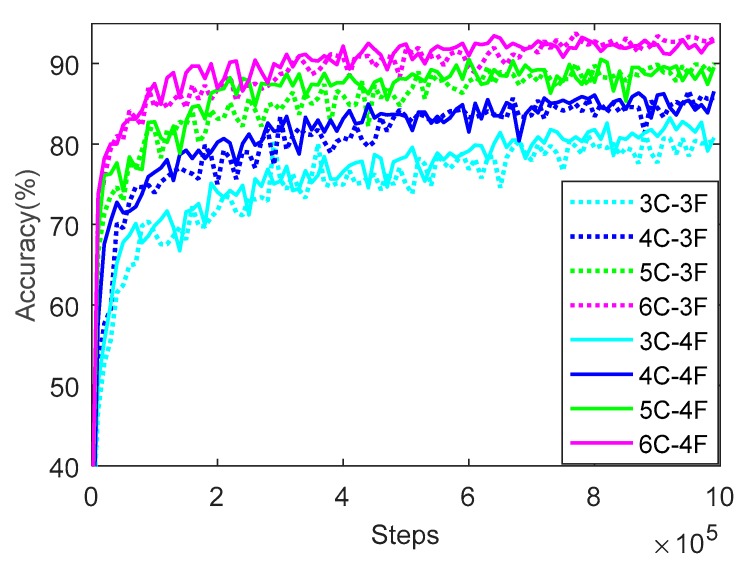
Prediction accuracy of CNN with different layers using the training set.

**Figure 7:**
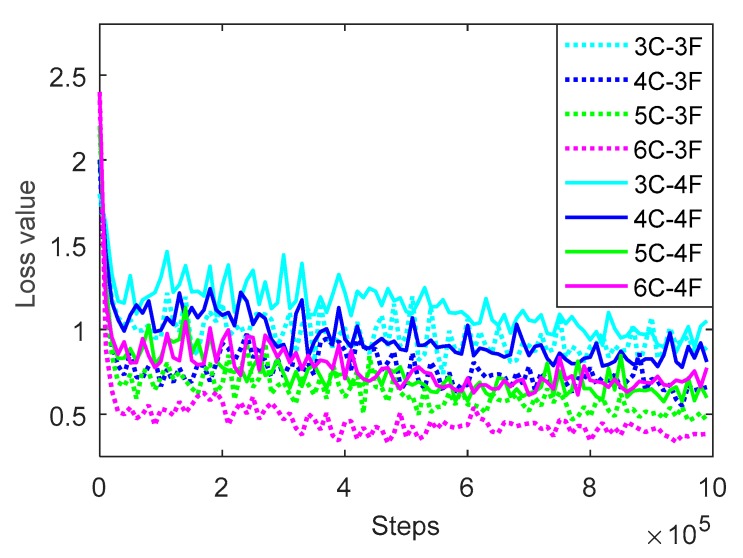
Loss value of CNN with different layers using the training set.

There are 10,696 samples in the training set, and the number of layers of CNN is tested. The network structures with convolution layers of 3, 4, 5, and 6, and full connection layers of 3 and 4 are trained, respectively. The results of prediction accuracy and loss value on the training set are shown in Figure 6 and Figure 7, respectively. The legend in the figure represents the network structure with different convolution layers and full connection layers. For example, 4C-3F represents the structure with 4 convolution layers and 3 full connection layers.

As seen in Figure 6, the dotted curve means the prediction accuracy of CNN with three full connection layers and the solid curve means that with four full connection layers, and different colors represent CNN with different number of convolution layers, among which cyan, blue, green, and pink represent the CNN with 3, 4, 5, and 6 convolution layers, respectively. Regardless of whether the full connection layer is 3 or 4, the prediction accuracy increases gradually with the increase of the number of convolutional layers, and shows a trend of rapid rise, gradual and slow increase, and then tends to be stable. When the number of convolutional layers is 3, 4, 5, and 6, the prediction accuracy reaches about 80%, 86%, 90%, and 93%, respectively, when training to 990,000 rounds. On the whole, when the convolutional network has 6 convolutional layers and 3 fully connected layers, the network obtains best prediction effect and achieves the highest prediction accuracy of 93.33% at 950,000 rounds.

As seen in Figure 7, the dotted curve means the loss value of CNN with three full connection layers and the solid curve means that with four full connection layers, and different colors represent CNN with different number of convolution layers, among which cyan, blue, green, and pink represent the CNN with 3, 4, 5, and 6 convolution layers, respectively. It can be seen that for the CNN with 3, 4, and 5 convolution layers, no matter there are 3 or 4 connection layers, the loss value shows the trend that falls down quickly firstly, then goes down slowly until to be steady, and the more layers, the lower the loss value. For the three kinds of network structure, the loss value is 0.96, 0.73, and 0.52 when the training runs to 990,000 rounds, respectively. However, for all kinds of network structure, the loss value of CNN with 3 connection layers is always smaller than that with 4 connection layers. Especially for the CNN with 6 convolution layers and 3 connection layers, it achieves the lowest loss value, and reaches the lowest value of 0.3369 when training runs to 940,000 rounds, which is 0.5742, 0.2521, and 0.1499 lower than the other three kinds of CNN with 3 connection layers, respectively. 

**Figure 8:**
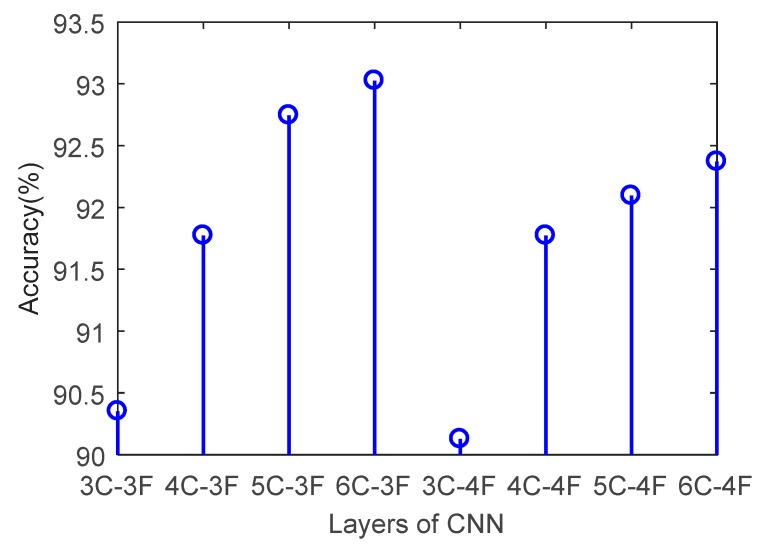
Validation on prediction accuracy with different layers using the testing set.

Convolutional networks with different layers are validated using the testing set, and the prediction accuracy obtained from the experiments is shown in Figure 8.

It can be seen from Figure 8 that the convolutional network with three full connection layers in testing set has higher accuracy than that with four full connection layers. When the network contains 3, 4, 5, and 6 convolutional layers, respectively, corresponding to 3 full connection layers, its recognition rate is 90.35%, 91.77%, 92.75%, and 93.03%, respectively, which is 0.22%, 0.65%, and 0.66% higher than that of the corresponding convolutional network with 4 full connection layers. The convolutional network achieves the highest recognition rate of 93.03% when contains 6 convolution layers and 3 full connection layers for the testing set.

The validation results using the testing set show that the network achieves the highest prediction accuracy and the lowest loss value when contains 6 convolution layers and 3 full connection layers, respectively. The prediction performance decreases when the number of full connection layers increases, which may lie in that the input is one-dimensional data with limited features, six convolution layers and three full connection layers are enough to extract all features from the input data, and any more layers will lead to overfitting. 

### 3.3. The Selection of Activation Function

Activation function is a key part of deep learning. Its function is to convert input from linear into nonlinear, which adds nonlinear factors to the network, so it is more expressive. After selecting the CNN with six convolution layers and three full connection layers, four different functions (sigmoid, tanh, ReLU, and Leaky ReLU) were tried for activation function selection. Xavier initialization method is applied with Keras for the activation functions of Sigmoid and tanh, and MSRA initialization method is applied with Tensorflow for the activation functions of ReLU and Leaky ReLU in this study.

The experimental results of the accuracy and the time to run a round under different activation functions were recorded, which can be shown in Table 3.

It can be seen from Table 3 that the tanh function has higher prediction accuracy than the sigmoid function (82.37% and 79.24%, respectively). This is because the sigmoid function maps input to the interval of [0, 1], and it tends to saturation when the value of sigmoid neuron is 0 or 1, and therefore will lose sensitivity when the value of sigmoid neuron is close to or exceed the interval of [0, 1], which makes the gradient in back propagation be zero and it no longer makes sense to update parameters with gradient descent method. In addition, it will cause most neurons saturated if weight is too large after initialization, as a result, the network stops learning, which affects the prediction accuracy. However, although the tanh function also has the problem of saturation and loses its sensitivity once it exceeds the corresponding interval, its output interval is [−1, 1], which is zero-centered, it has a better effect than sigmoid.

The ReLU function has the highest prediction accuracy (93.78%), which is because it is not saturated for positive numbers and hard saturated for negative numbers, which makes the output of neurons with negative input be zero. Therefore, the network sparsity is formed, the mutual dependence between parameters is reduced, and the overfitting problem is alleviated. Meanwhile, the training time of one round for the ReLU function is 0.4523 seconds, which is nearly six times faster than sigmoid and tanh functions (2.7256 and 2.7361, respectively). This is because the latter two functions need to perform an exponent calculation which takes long time, and also the calculation of error gradient by back propagation involves division operation and this calculated amount is very large too. However, ReLU function only needs one threshold to get the activation value, so the computing speed is faster. Meanwhile, the gradient descent algorithm convergence speed is faster too as it just involves simple linear calculation. 

The Leaky ReLU function is theoretically improved by the ReLU function to avoid hard saturation for negative area but gives a small gradient (0.01) for the negative value, although it solves the problem of dead neurons caused in the ReLU function, the effect is not stable in practical application, so the prediction accuracy is 3.52% lower than that of the ReLU function, and the running time is 0.0491 seconds longer. 

In summary, the ReLU function has the best performance on the whole. The reason why there is not much neuronal necrosis may be related to the reasonable setting of learning rate.

### 3.4. Results of Network Optimization

In this paper, batch standardization, L2 regularization and dropout technology are adopted to improve the generalization performance of the network. To avoid gradient disappearance or gradient explosion, the initialization method of MSRA for weights of each layer of the network is adopted too. 

**Figure 9:**
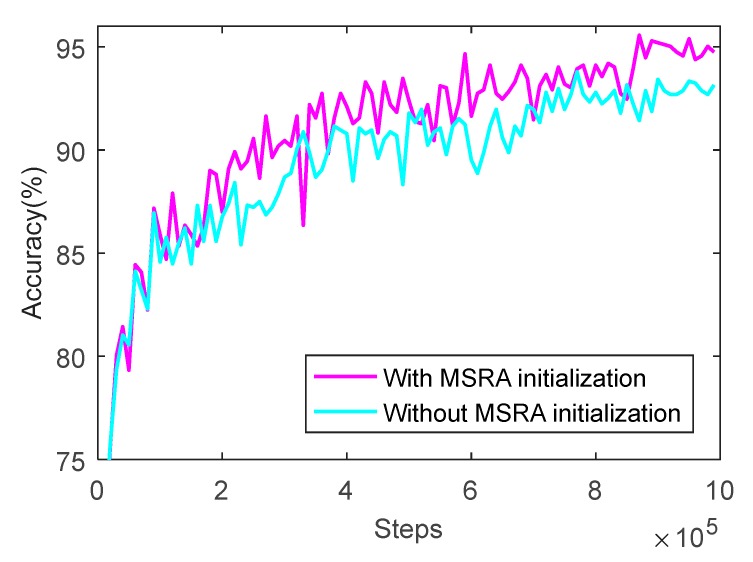
Prediction accuracy of CNN with MSRA (Microsoft Research Asia) initialization for weights using the training set.

**Figure 10:**
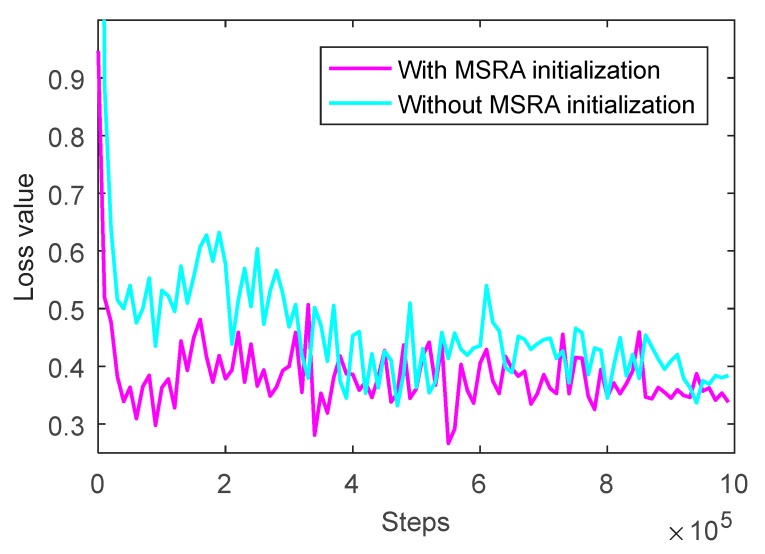
Loss value of CNN with MSRA initialization for weights using the training set.

The results of prediction accuracy of the network before and after MSRA initialization at different training times are shown in Figure 9, and the results of loss value are shown in Figure 10.

As can be seen from Figure 9, the prediction accuracy before and after optimization rises rapidly along with the training progress first, increases slowly, and finally trends to be steady state. However, after initializing the weight of the convolution kernel, the prediction accuracy of the network is significantly improved and it reaches 95.57% when the training runs 880,000 rounds, which is 4.14% higher than that before optimization.

As can see from Figure 10, the loss values before and after optimization drop rapidly, and then decrease slowly to reach a steady state. However, after initializing the weight of the convolution kernel, the loss value of the network significantly reduces on the whole, especially in the training process of the first 300,000 rounds, and it decreases by 0.2375 when the training runs to 260,000 rounds. The loss value tends to be stable along with training, and the loss value after optimization decreases to 0.2664, which is 0.1475 lower than that before optimization.

### 3.5. Simulation Results of the Optimized CNN Model

**Figure 11:**
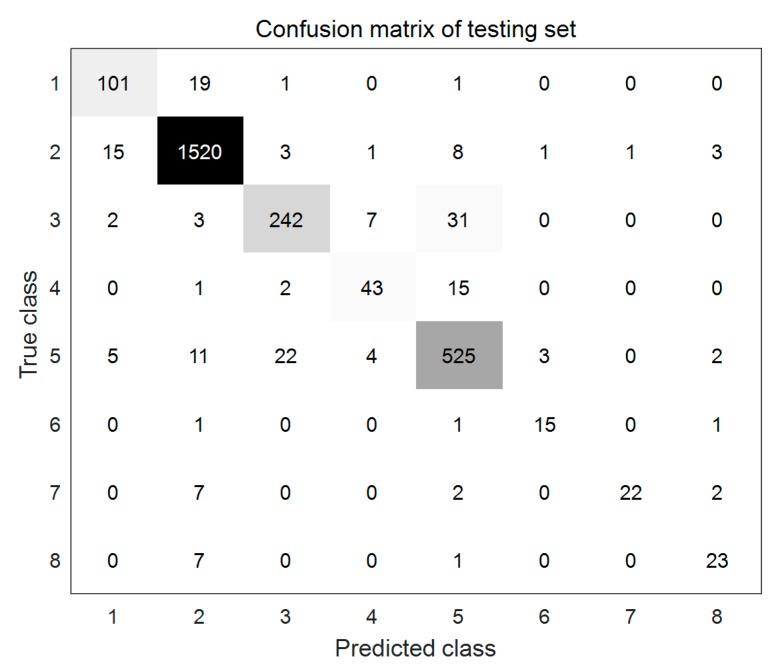
Confusion matrix of testing set.

After selecting the structure of CNN with 6 convolution layers and 3 connection layers, the simulation was conducted for the testing set with the optimized model, and the optimized model was further evaluated by the evaluation parameters of $\gamma_{TP}$, $\gamma_{TN}$, $\gamma_{PP}$, and γ. The mixture matrix for the testing set is shown in Figure 11. The values on the diagonal line of the figure are the number of correct predictions for samples, while the values on the off-diagonal line are the number of wrong predictions for samples.

Analysis of the above experimental results shows that, after the weight of convolution kernel is initialized with MSRA, the recognition rate of the network is significantly improved and the loss value is significantly reduced, which improves the prediction performance of the network and obtains a good optimization effect.

The experimental evaluation results are shown in Table 4. Larger values of the evaluation parameters in the table mean better prediction performance. It can be seen from Table 4 that when the sample number is over 100, the more samples, the higher the prediction accuracy $p_{a}$ (the prediction accuracy is 97.94%, 91.78%, 86.91%, and 85.79% respectively, corresponding to the sample number of 1552, 572, 285, and 122). However, the prediction accuracy is below 85.79% when sample size is lower than 100, and they are not no longer proportional. For example, the prediction accuracy is as high as 85.33% with 18 samples but it is as low as 68.67% with 33 samples. 

As the prediction accuracy of samples with negative labels is very high, the specificity rate for all samples is over 0.97. The case of the other evaluation parameters is similar to the case of prediction accuracy.

To compare with shallow network and traditional classification algorithm in terms of prediction accuracy, SVM, GA-SVM, and ANN models are used to be trained. These four models are used to test for two different data sets, and the results are given in Table 5. It can be seen from the table that for all four models, the prediction accuracy increases along with the increase of sample number. For two different datasets, the ANN model always gets the lower prediction accuracy than SVM and GA-SVM, which is because the ANN is a simple network with three layers and it is unable to adequately express information of high-dimensional data, but SVM is better at dealing with data with nonlinear relationships because of the kernel function. However, for CNN model, the prediction accuracy for small dataset (62.31%) is far lower than that of the other three algorithms, which is 18.06%, 21.07%, and 17.03% lower than SVM, GA-SVM, and ANN, respectively, but the prediction accuracy for large dataset (93.16%) is higher than that of the other three models, which is 6.79%, 2.48%, and 7.82% higher than that of SVM, GA-SVM, and ANN, respectively. This is because CNN model is a deep network, which is more suitable for the high-dimensional large sample data, but not good for small sample data, as small sample data cannot meet the needs of deep network that has many weights to be tuned by samples. SVM or GA-SVM, in comparison to CNN, is more suitable for high dimensional small sample data. Therefore, CNN shows better performance than SVM when the amount of data is large, but it is inferior to SVM or GA-SVM for small sample data. The CNN model used in this study achieves higher prediction accuracy (93.16%) than the methods of X-ray fluorescence based on discriminant analysis (90.5%) [43], SVM model based on NIR data (91.03%) [44], and TQ analysis software (90%) [45], which demonstrates again that CNN can be used for cultivation region classification of tobacco cultivation region.

To compare the computational cost of the deep CNN with traditional methods and the shallow network, the training time using different methods for the training set of big sample data (13370) was recorded, and the result is given in Table 6. It shows that CNN requires the longest time (6833.06 seconds), which is much longer than other methods. This is because the CNN model in this study is a deep network with six convolution layers and three fully connected layers, and it also has complex internal structure and a large number of hyperparameters, which makes the training process extremely time consuming. On the other hand, the traditional methods (SVM and GA-SVM) and the shallow network (ANN) have less complex internal structure and a smaller number of hyperparameters than CNN, thus need less training time.

## 4. Conclusions

In this paper, an improved deep CNN based on BN and MSRA initialization is developed to discriminate the tobacco cultivation regions using the data collected from NIR sensors. The CNN with six convolution layers and three connection layers is determined by experiments, and the exponential attenuation method is applied to control the learning rate. Four different activation functions are tested, the experimental results are analyzed and compared, and the ReLU function is finally selected according to the analysis of experimental results. The cross-entropy function is used as the loss function in this algorithm, and methods such as L2 regularization and dropout technology are adopted to avoid the overfitting problem and to improve the generalization ability of the network. 

To further optimize the network and avoid the gradient disappearance and gradient explosion phenomenon, the MSRA initialization method is applied for weights of each layer in the network. Experimental results show that the convolution kernel with one-dimensional vector can extract the features from complex characteristic information from the NIR data and the CNN can be used for cultivation region classification of tobacco. The results also demonstrate that the optimized CNN model can significantly improve the prediction accuracy and the deep network structure of CNN can be effectively used for big data analysis, and its good recognition effect has a practical value.

## Figures and Tables

**Table 1:** Regional distribution of all samples collected.

Class	Region	Number of Samples
1	West	557
2	Northwest	7668
3	Northeast	1422
4	Southeast	326
5	North	2897
6	South	132
7	Middle	164
8	Southwest	204

**Table 2:** The confusion matrix.

		Predicted Label	
		Positive	Negative
**Given Label**	**Positive**	$n_{TP}$	$n_{FN}$
	**Negative**	$n_{FP}$	$n_{TN}$

**Table 3:** Selection of activation function.

Activation Function	$p_{a}$	Time (s)
Sigmoid	79.24%	2.7256
Tanh	82.37%	2.7361
ReLU	93.78%	0.4523
Leaky ReLU	90.26%	0.5014

**Table 4:** Identification capacity of CNN for testing set on terms of five evaluation parameters. ($p_{a}$: prediction accuracy; $\gamma_{TP}$: sensitivity rate; $\gamma_{TN}$: specificity rate; $\gamma_{PP}$: precision rate; γ: F1-score).

Region	Number of Samples	$p_{a}$	$\gamma_{TP}$	$\gamma_{TN}$	$\gamma_{PP}$	γ
West	122	85.79%	0.8279	0.9914	0.8211	0.8245
Northwest	1552	97.94%	0.9794	0.9563	0.9688	0.9740
Northeast	285	86.91%	0.8491	0.9883	0.8963	0.8721
Southeast	61	74.49%	0.7049	0.9954	0.7818	0.7414
Nirth	572	91.78%	0.9178	0.9719	0.8990	0.9083
South	18	85.33%	0.8333	0.9985	0.7895	0.8108
Middle	33	68.67%	0.6667	0.9996	0.9565	0.7857
Southwest	31	76.19%	0.7419	0.9970	0.7419	0.7419

**Table 5:** Comparison of prediction accuracy with four models for two different datasets.

Dataset	SVM	GA-SVM	ANN	CNN
500	80.37%	83.38%	79.34%	62.31%
13370	86.37%	90.68%	85.34%	93.16%

**Table 6:** Training time with different algorithms.

Algorithm	Traing Time (s)
SVM	3068.63
GA-SVM	4122.04
ANN	4091.18
CNN	6833.06
